# A Case Report to Reflect on the Origins of MMRd Mesonephric-like Ovarian Adenocarcinoma: Can It Be Defined as a Mϋllerian Neoplasm?

**DOI:** 10.3390/ijms26115245

**Published:** 2025-05-29

**Authors:** Nicoletta D’Alessandris, Angela Santoro, Michele Valente, Giulia Scaglione, Giuseppe Angelico, Belen Padial Urtueta, Nadine Narducci, Simona Duranti, Francesca Addante, Angelo Minucci, Gian Franco Zannoni

**Affiliations:** 1 Pathology Unit, Department of Woman and Child’s Health and Public Health Sciences, Fondazione Policlinico Universitario Agostino Gemelli IRCCS, 00168 Rome, Italy; nicoletta.dalessandris@policlinicogemelli.it (N.D.); angela.santoro@policlinicogemelli.it (A.S.); michele.valente@guest.policlinicogemelli.it (M.V.); giulia.scaglione@policlinicogemelli.it (G.S.); belen.padialurtueta@policlinicogemelli.it (B.P.U.); nadine.narducci@policlinicogemelli.it (N.N.); francesca.addante@policlinicogemelli.it (F.A.); 2 Pathology Institute, Catholic University of Sacred Heart, 00168 Rome, Italy; 3 Department of Medicine and Surgery, Kore University of Enna, 94100 Enna, Italy; giuseppe.angelico@unikore.it; 4 Scientific Directorate, Fondazione Policlinico Universitario Agostino Gemelli IRCCS, 00168 Rome, Italy; simona.duranti@policlinicogemelli.it; 5 Departmental Unit of Molecular and Genomic Diagnostics, Fondazione Policlinico Universitario A. Gemelli IRCCS, 00168 Rome, Italy; angelo.minucci@policlinicogemelli.it

**Keywords:** ovarian cancer, mesonephric adenocarcinoma, MMR, Mϋllerian

## Abstract

Mesonephric-like adenocarcinoma (MLA) of ovaries is a new and rare neoplastic entity, recently classified by the World Health Organization. Its morphological and immunohistochemical profile is similar to primitive cervical mesonephric adenocarcinoma, but its origin has not been determined yet. Some authors believe that this neoplasm originates from Wolffian remnants in the ovarian hilum, while others suggest an origin from the Mϋllerian epithelium, followed by a mesonephric trans-differentiation. Starting from a recently diagnosed mismatch repair-deficient ovarian MLA, we try to further develop this line of research. A detailed molecular analysis of the studied tumor helps clarify our ideas. In fact, the typical KRAS mutation was not present. We found mutations in numerous other genes, which are rarely described in the literature or are already described in the endometrioid histotype. We reached some interesting conclusions, which, if supported by future studies, will clarify the true nature of these tumors, allowing for better stratification and a better therapeutic framework.

## 1. Introduction

Mesonephric adenocarcinoma (MA) is a rare malignant tumor of the female genital tract that includes less than 1% of all gynecological malignancies. It is thought to arise from the embryonal remnants of the Wolffian tubules and ducts, which regress during normal female development. MA, like mesonephric remnants, is typically located in the uterine cervix and/or vagina. However, several cases of malignant mesonephric lesions arising in the uterine corpus, vagina, and adnexa have also been reported [1]. Within this context, MA of the upper female genital tract has been referred to as mesonephric-like adenocarcinoma (MLA) because the association with mesonephric remnants has not been firmly established [2,3].

MLA was introduced as a new tumor type in the 2020 World Health Organization (WHO) Classification, both in the endometrium and in the ovary. Although they show significant morphological, immunophenotypic, and molecular overlap with cervical mesonephric adenocarcinomas, there are other parameters that suggest a Mϋllerian origin and bring them closer to endometriosis-related ovarian cancers, such as endometrioid and clear cell ones [4]. According to the TCGA (The Cancer Genome Atlas)’s molecular classification of endometrial carcinomas, they would belong to the copy number low/no specific molecular profile category. KRAS and PIK3CA mutations are the most common genetic alterations encountered in MLA [5,6,7].

On this wave and based on Mirkovic et al.’s work, in January 2024, we described the case of a 59-year-old woman with MLA of the endometrium showing a surprising MMR (mismatch repair) deficiency, trying to explain the possible pathogenesis of these aggressive tumors. Are they Mϋllerian or mesonephric tumors [8,9]? One year later, our group found ourselves diagnosing a similar case (MMR-deficient MLA), but in the ovary. Thus, we asked ourselves the same question, reaching more convincing conclusions.

## 2. Materials and Methods

**Figure 1:**
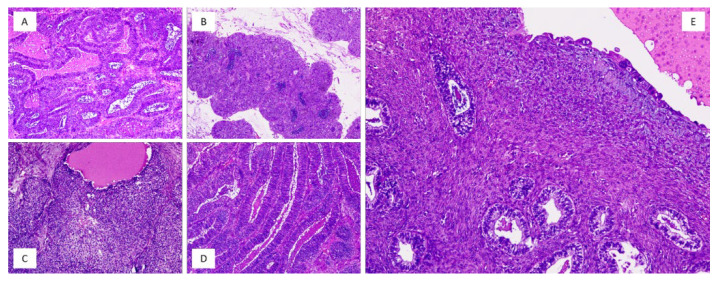
Histological appearance of the neoplasm. Ovarian carcinoma showed an endometrioid component ((**A**,**D**)—hematoxylin and eosin, 20× magnification) characterized by glandular pattern, and a mesonephric-like component ((**B**,**C**)—hematoxylin and eosin, 20× magnification) characterized by solid growth in the picture, with scattered accumulation of eosinophilic colloid-like material, and elongated nuclei with occasional grooves. In (**E**) (hematoxylin and eosin, 10× magnification), a detail of the neoplasm showed the precursor lesions: endometriotic cyst with metaplastic changes (top) and endometrioid borderline tumor (bottom).

**Figure 2:**
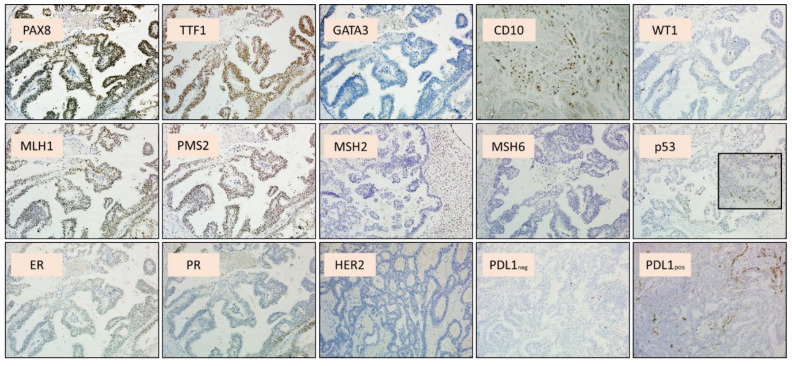
Detailed immunohistochemical profile of the neoplasm. Both the neoplastic components showed positivity with PAX8, TTF1 (diffuse and strong), CD10 (luminal and focal staining), ER (partial), and PR (partial), and negativity for GATA3 and WT1. P53 was wild-type; note the 40× magnification in the detail. MSH2/MSH6 expression was lost, and MLH1/PMS2 expression was preserved. HER2 was negative (score 0). PD-L1 was positive using SP142 clone—Ventana—and negative using 22C3 clone—DAKO.

The case concerns a 49-year-old woman with a family history positive for endometrial cancer (mother and grandmother). The patient reported pelvic pain for which she underwent a control ultrasound scan, revealing a left ovarian lesion suspicious for malignancy. Intraoperative examination showed high-grade ovarian cancer. So, she underwent a radical hysterectomy with the removal of bilateral ovaries and fallopian tubes. The ovary was replaced by an 8 cm solid mass covered by an intact capsule at the IB FIGO stage. Histological evaluation [Figure 1] showed a high-grade epithelial neoplasm with two different neoplastic components: high-grade endometrioid carcinoma (EC) and mesonephric-like adenocarcinoma. The predominant growth pattern was solid and glandular with eosinophilic luminal colloid-like material, admixed with papillary architecture and slit-like spaces, medium-sized glands or duct-like formations, and cystically dilated glands. Necrotic foci and numerous mitoses were present. Neoplastic cells were characterized by moderately atypical nuclei, with occasional nuclear grooves and hobnail appearance. Benign endometriosis foci were identified near the neoplastic proliferation. All these morphological features were suggestive of mesonephric-like differentiation. This dominant component was intermingled with glandular structures characterized by atypical cells with squamous metaplasia, resembling endometrioid histotype. The immunohistochemical study [Figure 2] confirmed the presence of a predominant MLA and a minor (10%) component of EC. The tumor exhibited positivity with PAX8, TTF1 (diffuse and strong in MLA), CD10 (luminal and focal staining in MLA), ER (partial, mostly in the endometrioid component), and PR (partial, mostly in the endometrioid component), and negativity for GATA3 and WT1. P53 was only focally positive, which means a wild-type phenotype. Interestingly, we observed (again) an MMRd (mismatch repair-deficient) profile: lost expressions of MSH2 and MSH6, and preserved expressions in MLH1 and PMS2, in both the neoplastic components. HER2 (4B5 clone—Ventana) was negative (score 0). PD-L1 (Programmed Death—Ligand 1) expression was positive (>1%) when using the SP142 clone—Ventana—and negative (<1%) when using the 22C3 clone—DAKO.

To further confirm our diagnosis and to better understand the differentiation of our tumor, we performed next-generation sequencing (NGS) analysis. We performed molecular analysis on a single FFPE block representative of both tumoral components. Molecular profiling was performed with the TruSight Oncology 500 high throughput (TSO500HT, Illumina) that analyzes both DNA and RNA, identifying single-nucleotide variants (SNVs), insertions/deletions (indels), and copy number variations (CNVs) in 523 genes, as well as known and unknown fusions and splicing variants in 55 genes and provides genomic “signatures” such as microsatellite instability (MSI) and tumor mutational burden (TMB). Using this procedure, we encountered several interesting results. Both the neoplastic components presented the same molecular alterations and were as follows: ATM [c.9022C>T (p.Arg3008Cys); NM_000051.3; VAF 26.3; Dept 346]; FBXW7 [c.745C>T (p.R249); NM_0010133415.1; VAF 22.9; Dept 249]; MSH2 [c.2536C>T (p.Q846); NM_000251.2; VAF 75.8; Dept 367]; NF1 [c.6951G>A (p.W2317); NM_001042492.2; VAF 35.7; Dept 182]; PIK3CA [c.1359_1361delAGA (p.E453del)—NM_06218.2; VAF 27; Dept 200-; c.3140A>G (p.H1047R)—NM_06218.2; VAF 26.9; Dept 275]; PPP2R1A [c.547C>T (p.R183W); NM_014225.5; VAF 25.1; Dept 1281]; MST1 [c.1423+2T>A p; NM_020998.3; VAF 15; Dept 635].

Our study was conducted in accordance with Good Clinical Practice guidelines and the Declaration of Helsinki (1975, revised in 2013). The clinical information had been retrieved from the patient’s medical records and pathology reports. Patient’s initials or other personal identifiers did not appear in any image. Finally, all samples were anonymized before histology and immunohistochemistry. Due to the retrospective nature of the study, our institution did not deem the approval of an ethical committee as necessary because the analyzed data were collected as part of routine diagnosis, and patients were diagnosed and treated according to national guidelines and agreements. Our analysis looked retrospectively at the treated patient outcomes. This was achieved internally as part of an audit/evaluation to improve our quality of care.

## 3. Results and Discussion

Gynecological mesonephric-like adenocarcinoma is a rare tumor type. It is known to everyone that morphology and immunohistochemistry are similar to mesonephric adenocarcinoma. The same cannot be said for their histological origin. Its molecular background is still under debate, and we are still in the preliminary phases. In a recent article, Lin et al., based on a previous work published by Kommoss et al., affirmed that MLAs more closely resemble cervical MA than Mϋllerian carcinomas on an epigenetic level [10,11]. On the other hand, along with those already mentioned, McCluggage and Mirkovic [6,7,8] are also more in favor of a Mϋllerian origin of the neoplasm. Poor prognosis and lack of specific therapeutic standards are major challenges of this disease. Having a clearer understanding of their differentiation line could perhaps help us in this sense.

We will begin our discussion by examining all the genes we found mutated in the studied tumor. We will try to summarize the role of each gene and its relationship with MLA, based on what is already known in the literature.

**ATM** (Ataxia-telangiectasia mutated) is a gene that functions as a key initiator and coordinator of DNA damage and cellular stress responses. It is known that ATM mutations are connected with increased breast cancer risk [12]. There have been two reported cases of malignant mesonephric lesions harboring ATM mutations, with conflicting interpretations of pathogenicity [13,14,15].

**FBXW7** (F-Box and WD Repeat Domain Containing 7) is a member of the F-box protein family and acts as a tumor suppressor gene. It has been observed to be frequently mutated in multiple human cancers, such as colorectal, ovarian [16], breast, and endometrial cancer [17]. FBXW7 is often somatically mutated in grade 3 endometrioid endometrial cancers and serous ECs [18].

**MSH2** (MutS Homolog 2) is a DNA repair gene encoding a DNA mismatch repair (MMR) protein. Mismatch repair deficiency is observed in 25–30% of all endometrial cancers. About 40% of high-grade endometrioid carcinomas belong to the MMRd TCGA group [19].

**NF1** (Neurofibromatosis Type 1) is a tumor suppressor gene located on chromosome 17; its product, the neurofibromin, is a negative regulator of the RAS-MAPK pathway. Mutations in the NF1 gene are commonly known to cause Neurofibromatosis type 1, an autosomal dominant disorder with multiorgan involvement (café-au-lait spots, skinfold freckling, Lisch nodules, neurofibromas, bone abnormalities, and optic pathway glioma). Regarding neoplastic pathologies and gynecological tumors, an association has recently been found between NF1 inactivation and a subset of ovarian cancers (breast cancer gene 1/2 wild-type high-grade serous carcinomas and low-grade serous carcinomas), giving a potential therapeutic opportunity for these patients [20]. We want to point out the case of a patient with NF1 who developed high-grade serous ovarian cancer, supporting the concept that females with NF1 should also be monitored for ovarian and breast cancers [21,22].

The identification of **PIK3CA** (phosphatidylinositol-4,5-bisphosphate 3-kinase catalytic subunit alpha) mutation in the present case is in line with previous observations [23,24]. Mirkovic et al. found PIK3CA mutations in 3 of 7 (43%) MLAs [8]. It is worth noting that PIK3CA is the second most frequently significantly mutated gene after PTEN in primary endometrial cancers, with a frequency of 53%. An association between exon9 mutations and poor outcome has been demonstrated [24].

**PPP2R1A** (Protein Phosphatase 2 Scaffold Subunit Alpha) is a gene implicated in the negative control of cell growth and division. Recently, it has been studied in endometrial cancer and considered a new promising biomarker and therapeutic target for these neoplasms (both type I and type II endometrial neoplasms) [25]. PPP2R1A mutations may be associated with tumor immune evasion, allowing tumor cells to escape immune surveillance and attack, increasing the risk of a poor prognosis. Patients with PPP2R1A-mutated endometrial cancer had longer OS with lenvatinib and pembrolizumab than those with wild-type PPP2R1A [26].

The **MST1** (Macrophage Stimulating 1) gene has an active role in immune regulation, inflammatory response, mitochondrial apoptosis, and autophagy in oxidative stress conditions. In the literature, the authors correlate this gene more to inflammatory and degenerative cardiovascular or cerebral diseases than to neoplastic events [27,28]. It is now known that MMRd endometrioid cancers are characterized by a high amount of intratumoral inflammatory cells, a feature that makes them amenable to immunotherapy [29]. Could the MST1 mutation be linked to intratumoral microenvironment and, more generally, to the MMRd histotype? However, a recent paper published in *Cancers* demonstrated that the human Hippo gene mammalian sterile 20-like kinases 1 (MST1), and its downstream target gene yes-associated protein 1 (YAP1), could predict the serous subtype ovarian tumors [30].

The **TMB** (Tumor Molecular Burden) was high, with a value of 52.56 Mut/Mb. Genetic counseling and germline testing for NF1 are ongoing. Germline MSH2 test revealed a constitutional pathogenetic nature.

Our case exhibited strikingly similar histological and immunohistochemical features to mesonephric adenocarcinomas, which supported our final report, but also showed typical molecular aberrations of endometrioid carcinomas, which fueled our doubts about the origin.

To our knowledge, this is the first time that FBXW7 and NF1 mutations have been reported in MLA. On the contrary, there are two previous cases in the literature reporting on ATM mutation [13,14] and one case reporting on PPP2R1A mutation [31]. PIK3CA and MSH2 are more thoroughly studied genes in MLAs [8,9,21,22]. The MST1 gene has so far been mostly linked to non-neoplastic pathologies, so little is known about its oncogenic potential [27,28]. Although the role of most of these genes is still unclear, we can note a biological overlap with neoplasms of Mϋllerian differentiation. FBXW7, MSH2, PIK3CA, and PPPR21A are all genes usually found altered in EC.

Finally, we are aware that not all mutations in cancer are biologically relevant, especially in the presence of MMR defects. In fact, the described tumor has accumulated a high number of somatic microsatellite (MS) insertions or deletions and single-nucleotide variants (SNV), due to the loss of normal mismatch repair (MMR) capacity. For this reason, we have chosen to highlight only variants with a clear clinical significance for the tumor but also associated with a syndromic disease. This does not clarify whether they are drivers or passengers, but they certainly could have clinical significance.

Returning to histology, as McCluggage stated, in the uterine corpus, MLAs arise from the endometrium and spread into the myometrium; none of these neoplasms involved the myometrium without endometrial involvement, as might be expected with a true mesonephric carcinoma arising from mesonephric remnants. Moreover, in the ovary, these neoplasms are often associated with endometriosis, and, in both the uterus and the ovary, they have not been associated with mesonephric remnants [4]. Our group recently described a case of endometrial MLA in which a potential preneoplastic lesion was reported for the first time, demonstrating a continuum from mesonephric-like metaplasia and atypical mesonephric-like endometrial hyperplasia to MLA [32].

## 4. Conclusions

In conclusion, in agreement with previous authors [8], we believe that MLA should be best regarded as being of a Mϋllerian origin. These tumors appear to be closer to endometrioid carcinomas and should be categorized into the group of endometriosis-related ovarian cancers. Our case and its unique molecular features support the hypothesis that ovarian MLA may represent not a Wolffian-type but a Mϋllerian-type carcinoma, with secondary mesonephric differentiation. In recent years, evidence has been accumulated that increasingly suggests this theory, but it is not yet sufficient to assert this conclusion with certainty. Finally, this is the first time that an ovarian MLA was identified in a Lynch syndrome. However, a larger case study and further research are needed to better understand the real prognostic impact of the described molecular alterations, in order to define the clinical outcome of these patients.

## Data Availability

Data is contained within the article.
